# Supplementary material for: Antagonistic odor interactions in olfactory sensory neurons are widespread in freely breathing mice
Source: Nat Commun. 2020 Jul 3;11:3350. doi: 10.1038/s41467-020-17124-5 (PMC7335155; doi:10.1038/s41467-020-17124-5)
Supplement: Supplementary file 1 — Supplementary Information [file 41467_2020_17124_MOESM1_ESM.pdf]

Antagonistic odor interactions in olfactory sensory neurons are widespread in freely breathing mice

Zak et al.

Supplementary Figure 1

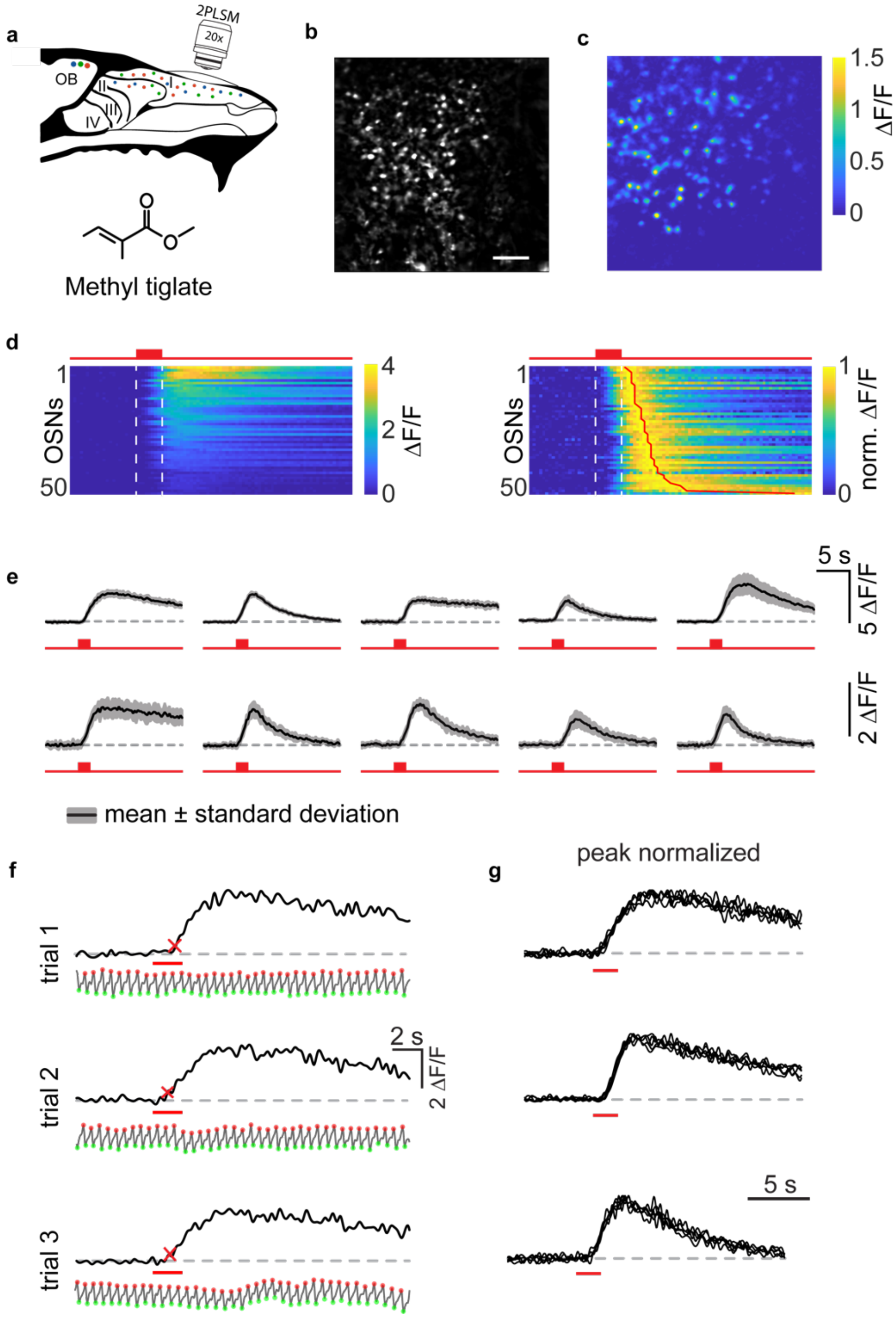

Response stability and kinetics of single OSNs.

a. Experimental setup for imaging OSN somata in the epithelium for the odor Methyl tiglate. **b**. Resting fluorescence of OSN somata in the olfactory epithelium. Scale bar in image is 50 μm. **c**. ΔF/F image in response to the odor Methyl tiglate for the imaging field in B. **d**. *Left*, time course of 50 selected OSNs sorted by response amplitude in response Methyl tiglate. *Right,* Same 50 OSNs normalized to their peak amplitude and sorted by peak position, indicated by the red vertical line. Color scale represents values normalized to each OSN peak response. Red box and dashed lines indicate odor delivery time. **e**. 10 selected OSNs showing odor response time course. Shaded area is standard deviation from 10 repetitions of odor delivery. **f**. Three consecutive responses from the same OSN and accompanying respiration trace. Peak of inhalation and exhalation are denoted by red and green circles. Odor delivery time is red bar. Red cross denotes point when trace first surpasses three standard deviations from baseline. **g**. Three example OSNs in response to three odor repetitions. Top traces correspond to part *e*. Traces are normalized to their peak amplitude to show waveform consistency across trials for the same odor.

Supplementary Figure 2

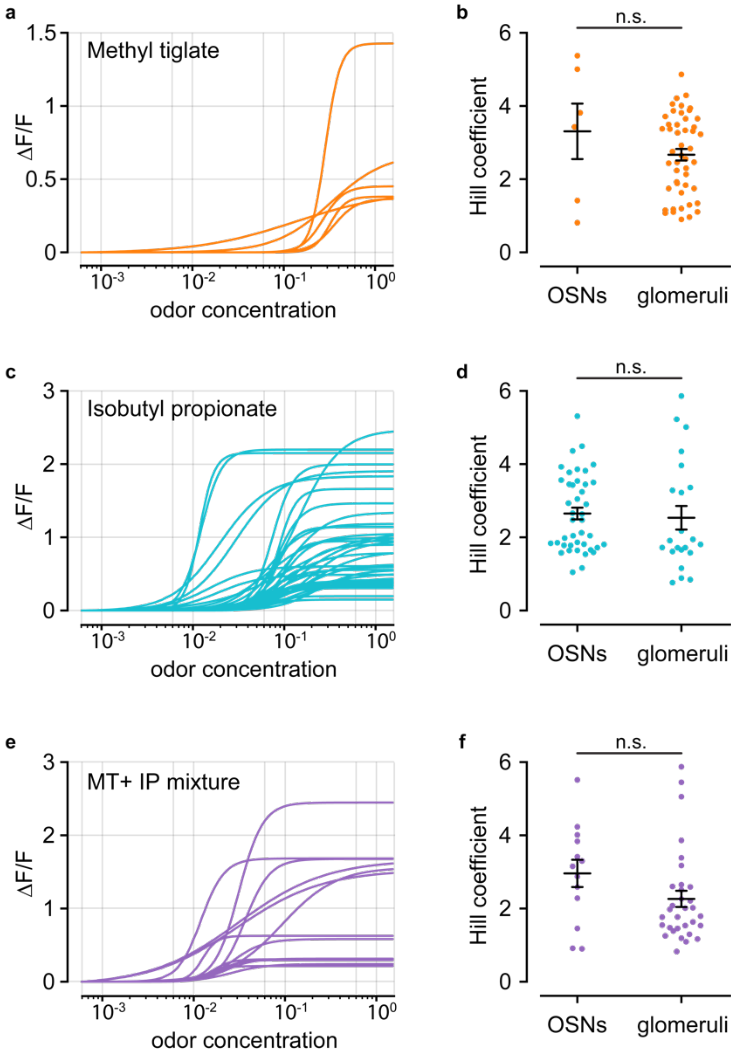

Hill coefficients of odor responses in individual OSNs.

a. Example response curve fits from individual OSN responses to Methyl tiglate. **b**. Hill coefficient distributions from the individual OSNs plotted in part *a* compared to the Hill coefficients from glomeruli for the same odor (OSN mean = 3.31 ± 0.76, n = 6 OSNs, glomerular mean = 2.67 ± 0.16, n = 48 glomeruli; $P > 0.05$, *Rank-sum test;* Data are presented as mean ± SEM). **c**. Example response curve fits from individual OSN responses to Isobutyl propionate. **d**. Hill coefficient distributions from the individual OSNs plotted in part *c* compared to the Hill coefficients from glomeruli for the same odor (OSN mean = 2.65 ± 0.16, n = 42 OSNs, glomerular mean = 2.53 ± 0.32, n = 22 glomeruli; $P > 0.05$, *Rank-sum test;* Data are presented as mean ± SEM). **e**. Example response curve fits from individual OSN responses to Methyl tiglate and Isobutyl propionate mixtures. **f**. Hill coefficient distributions from the individual OSNs plotted in part *e* compared to the Hill coefficients from glomeruli for the same mixture (OSN mean = 2.65 ± 0.16, n = 42 OSNs, glomerular mean = 2.53 ± 0.32, n = 32 glomeruli; $P > 0.05$, *Rank-sum test;* Data are presented as mean ± SEM).

Supplementary Figure 3

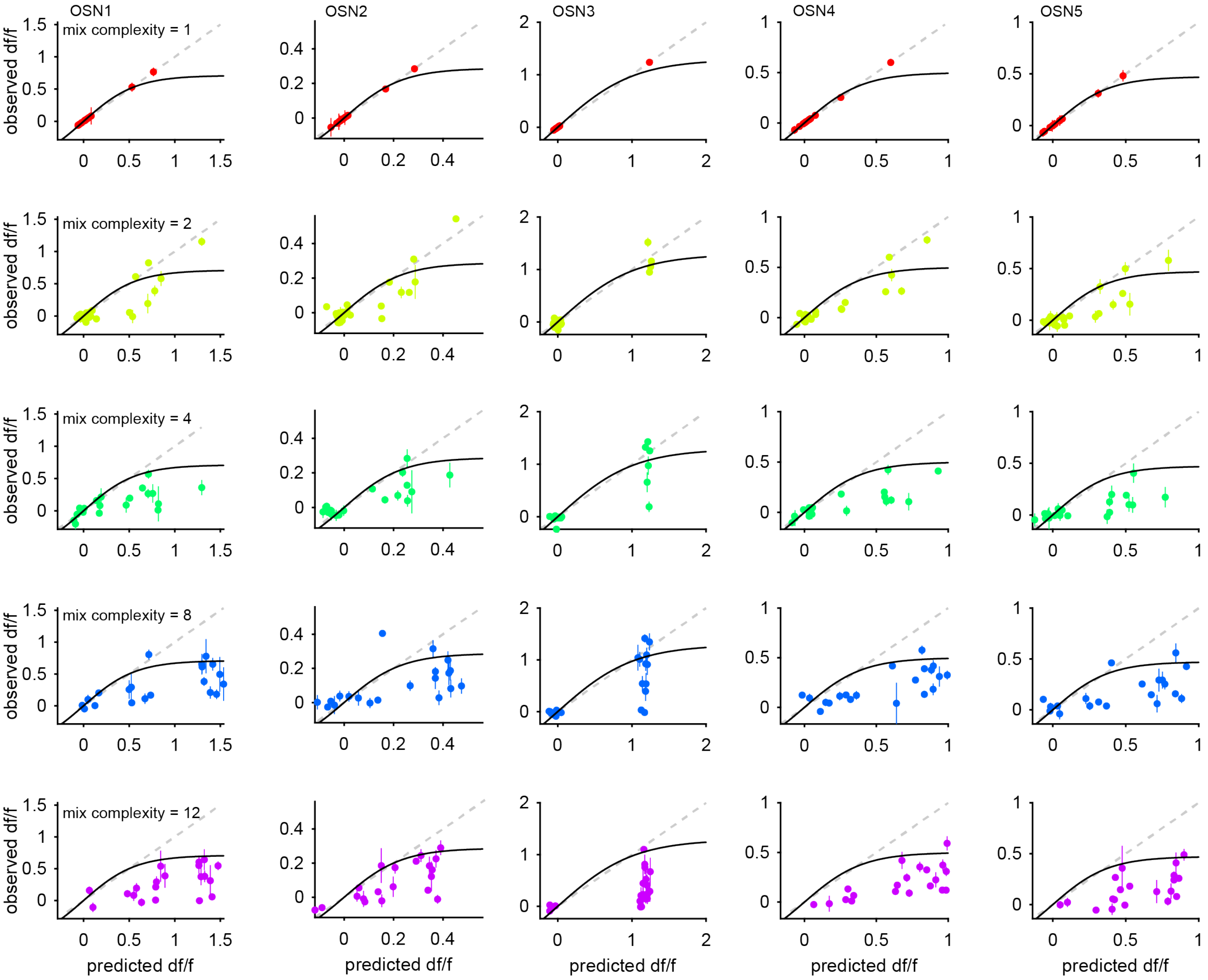

Mixture responses in individual OSNs. Related to Figure 7

Mixture responses for five selected OSNs. Each column corresponds to a single OSN and rows are mixture complexities arranged by increasing mixture complexity. Each point is presented as the mean ± SEM. of three repetitions of an odor mixture. Supplementary OSN1 corresponds to Figure 5 OSN2 and supplementary OSN3 corresponds to Figure 5 OSN4.

Supplementary Figure 4

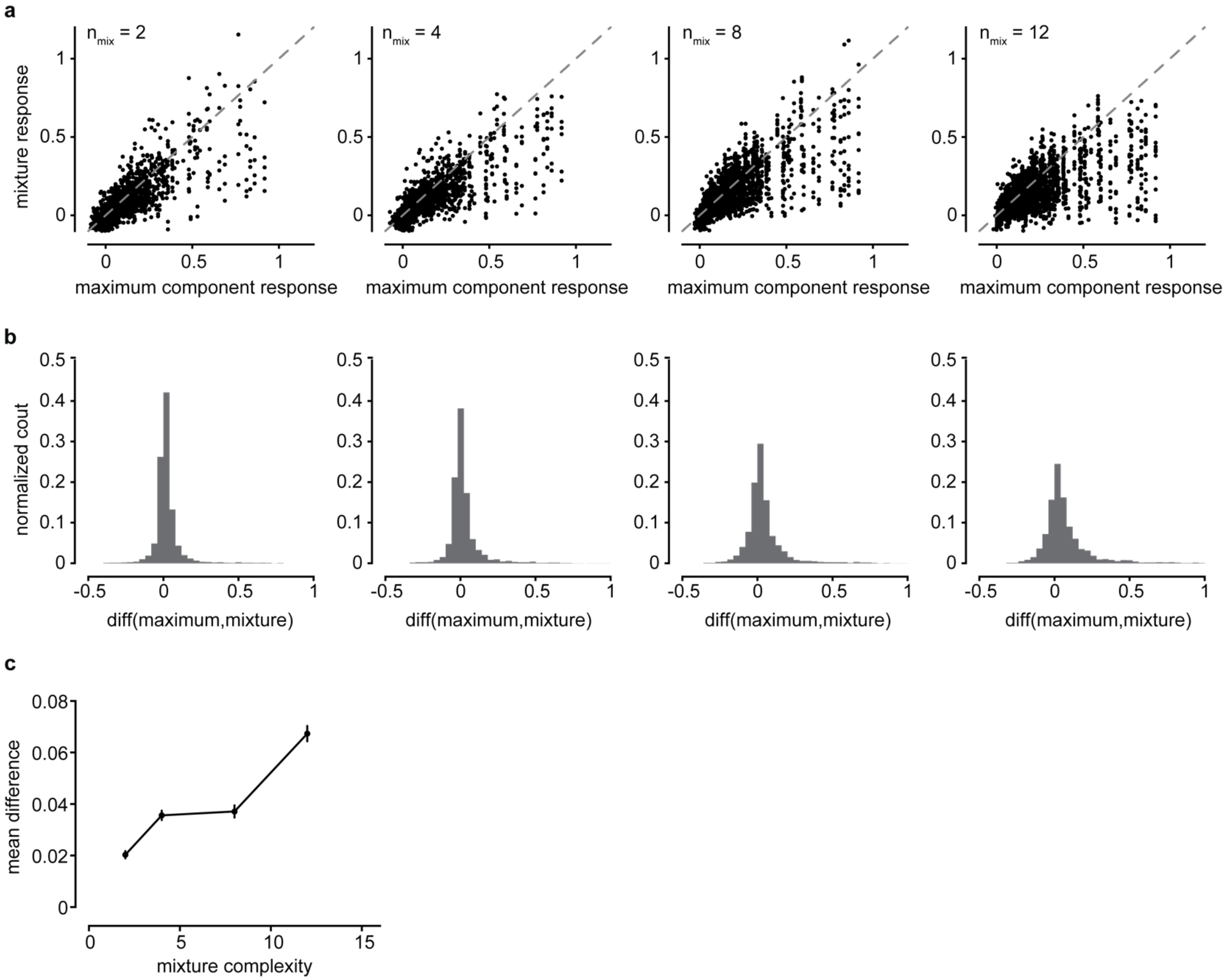

Odor mixture responses in OSNs compared to the maximum component response. Related to Figure 7.

a. Each mixture response is compared to the maximum response generated by any of the individual components included in the mixture. See Supplementary Table 1 for mixture compositions (n = 1800, n = 1596, n = 1861, n = 1948 OSN-odor mixture pairs, for 2-, 4-, 8-, and 12-part mixtures respectively). **b**. Differences between the maximum component response and the observed mixture response are plotted as density normalized histograms. Positive values indicate the maximum component response was larger than the mixture response. Note the reduction in central peak as mixture complexity increases. For each mixture complexity, the number of data points is consistent with part a. **c**. Mean difference across all observations for each mixture complexity. Error bars are s.e.m. The difference increases with mixture complexity. For each mixture complexity, the number of data points is consistent with part a.

Supplementary Figure 5

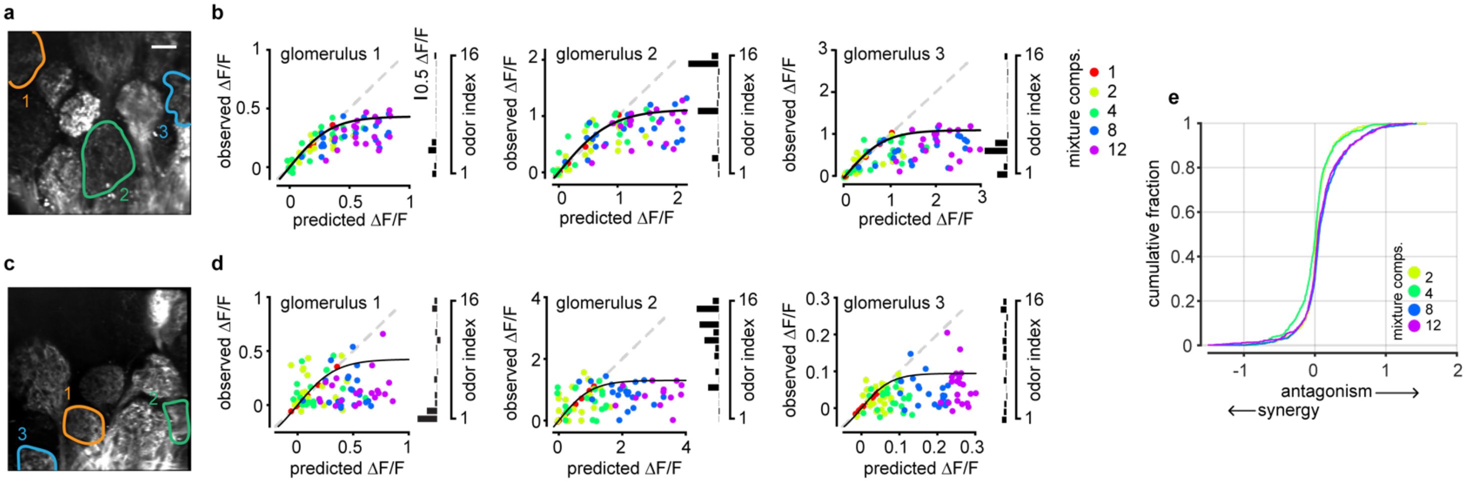

Complex mixture responses in OSN axon terminals. Related to Figure 5

a. Example image of glomeruli and selected ROIs. Scale bar is 50 µm. **b**. Example mixture responses from the glomeruli outlined in *a*. Each point is the average of three trials. The data from each glomerulus is fit with a sigmoid that asymptotes at the top 0.05 quantile of all responses. The odor tuning profile for each glomerulus is shown on the right. **c-d.** Same as a-*b*, but for another glomerular imaging field**. e**. Cumulative distribution of all deviations from the sigmoidal fit for each mixture complexity. Data collected from 39 glomeruli in four mice.

Supplementary Figure 6

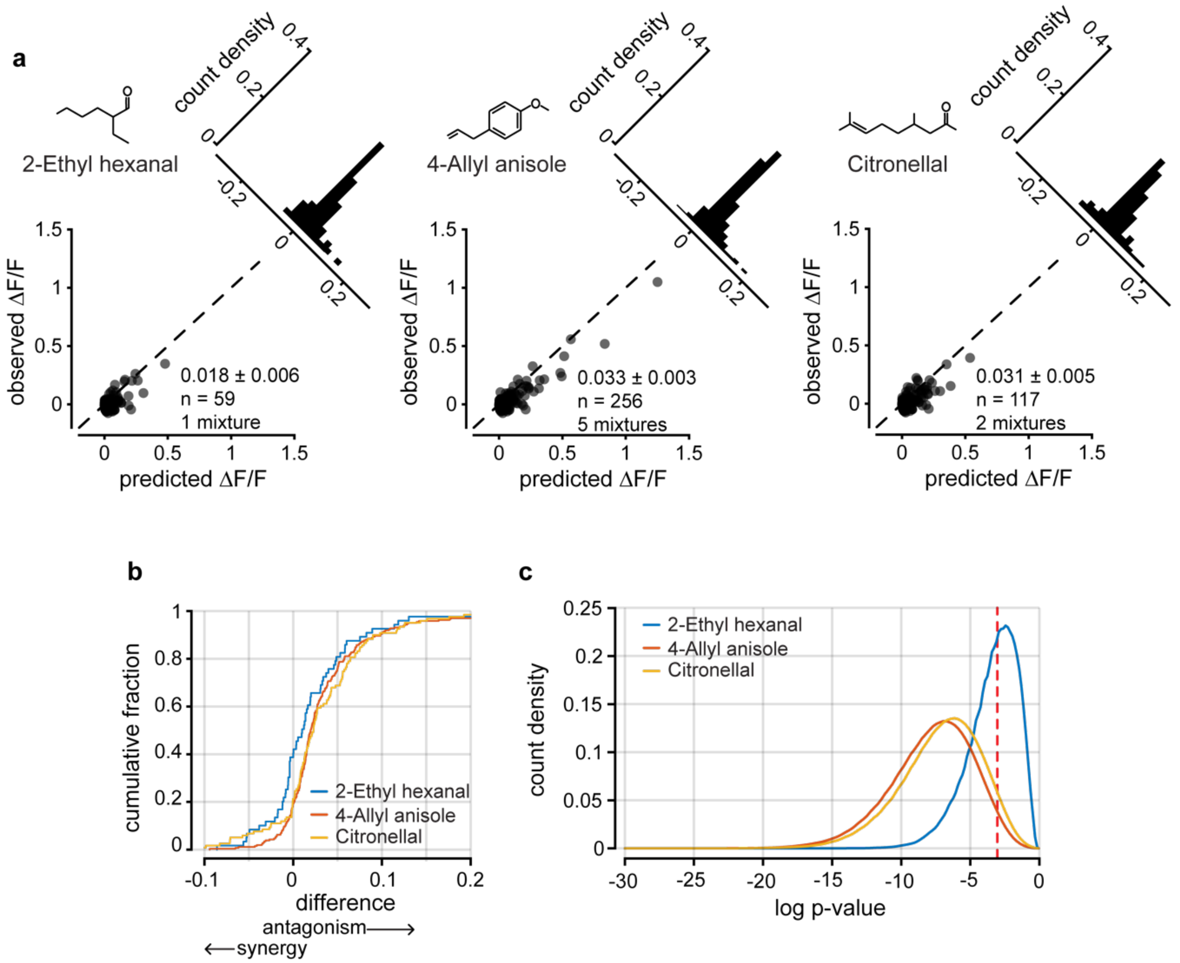

Binary odor mixtures with ester and non-ester functional groups

a. OSN responses to binary mixtures that contained one odor with non-ester functional groups (2-Ethyl hexanal, n = 59 OSN-mixture pairs; 4-Allyl anisole, n = 256 OSN-mixture pairs; Citronellal, n = 117 OSN-mixture pairs) and other odors containing an ester. Data are extracted from experiments in *Figure 5*. Predicted responses are the linear sum of each component alone. For each non-ester odor, the mean difference between the predicted and observed response is given. Positive values indicate mixture suppression. **b**. Cumulative distribution of the difference between the prediction and observation for each OSN-mixture pair for each of the three non-ester odors in *a*. **c**. Bootstrapped *P*-value distributions for each odor, testing the significance of observed differences in *b* from a null distribution. 50 random values from the distributions in *b* were selected and compared to distributions with the same standard deviation with a mean of zero using a *Kolmogorov-Smirnov* test. This process was repeated 100,000 times. Red vertical dashed line corresponds to *P*-values = 0.05.

Supplementary Figure 7

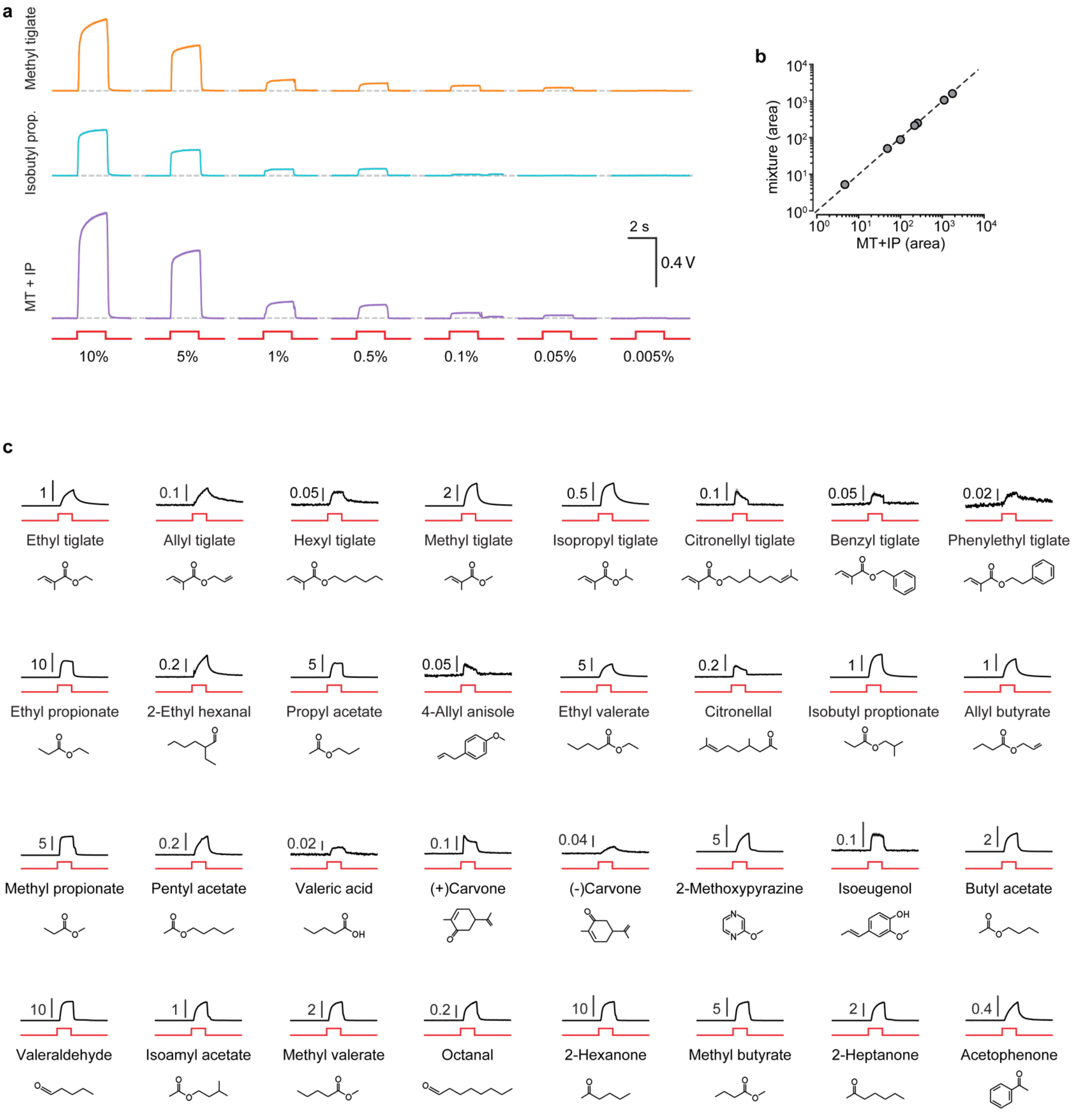

Photoionization detector (PID) traces for binary odor mixtures and complex mixture components

a. PID traces for the odor pair Methyl tiglate and Isobutyl propionate at seven v/v dilutions. Solid lines are mean of five trials and shaded area is SEM. Odors were delivered for 2 s. Voltage command to the olfactometer is in red. Estimated concentration after air dilution is given for each trace below. **b.** For each v/v dilution the linear sum of the area under the PID trace for each odor component is compared to the measured PID measured mixture response. The mixture response is nearly a perfect summation of mixture components when delivered alone. **c**. PID traces of all odors used in tuning experiments in black (mean of 5 trials), voltage command to olfactometer in red. Molecular shape of each odor is below the corresponding traces. Scale bar units are volts.

Supplementary Table 1

mixture size	mixture component indices	mixture size	mixture component indices
2	13,15	8	2,5,6,7,10,12,14,15
2	5,12	8	1,2,6,7,8,10,11,14
2	4,8	8	1,4,5,9,10,11,12,15
2	1,15	8	1,4,5,6,9,10,11,16
2	1,12	8	1,6,10,11,12,13,14,16
2	2,3	8	1,4,6,8,9,10,12,14
2	9,11	8	1,2,3,4,7,9,11,15
2	1,4	8	5,6,7,8,10,11,14,16
2	6,9	8	1,2,3,5,6,8,11,15
2	4,13	8	1,6,8,10,12,13,14,16
2	3,4	8	1,3,4,5,9,10,12,16
2	13,14	8	2,3,6,7,9,12,13,15
2	9,15	8	1,4,9,10,11,14,15,16
2	3,11	8	1,4,5,8,10,11,12,14
2	7,12	8	3,5,6,9,10,13,15,16
2	5,15	8	1,2,3,4,9,11,13,16
2	8,15	8	2,3,4,7,8,10,11,15
2	12,14	8	2,3,4,5,6,12,14,16
2	2,6	8	1,4,5,7,12,14,15,16
2	1,7	8	2,3,5,6,9,10,11,16
2	10,16	12	2,4,7,8,9,10,11,12,13,14,15,16
2	3,12	12	2,3,4,6,7,8,9,10,11,12,14,16
2	1,16	12	1,3,4,5,6,7,9,10,11,13,14,15
2	4,11	12	1,3,4,6,7,8,9,10,12,13,15,16
4	3,4,10,11	12	1,2,3,4,6,8,9,10,12,14,15,16
4	1,2,4,6	12	1,2,3,5,8,9,11,12,13,14,15,16
4	4,5,6,14	12	1,2,3,4,6,9,11,12,13,14,15,16
4	4,6,10,13	12	1,3,4,5,6,7,8,10,11,12,13,15
4	6,13,14,15	12	1,4,5,6,7,9,11,12,13,14,15,16
4	1,5,6,8	12	2,3,5,6,7,8,9,10,11,12,13,16
4	3,7,10,14	12	1,2,3,4,6,7,8,9,11,12,14,15
4	9,10,11,12	12	1,2,3,4,5,7,8,9,11,12,14,15
4	2,3,4,14	12	1,2,3,4,5,6,7,8,11,12,13,15
4	3,10,14,15	12	3,4,6,7,9,10,11,12,13,14,15,16
4	2,8,15,16	12	1,3,5,6,7,9,10,11,12,13,14,15
4	7,8,11,12	12	1,2,3,5,6,8,10,12,13,14,15,16
4	2,4,12,14	12	1,2,5,6,7,8,9,10,12,14,15,16
4	5,6,9,11	12	2,3,4,5,7,8,10,11,12,13,15,16
4	9,11,12,16	12	3,4,6,7,8,9,10,11,12,13,14,15
4	1,2,3,12	12	3,5,6,7,8,9,11,12,13,14,15,16
4	8,12,14,15		
4	3,4,8,9		
4	2,9,10,11		
4	3,8,9,13		

Odor mixture compositions

Related to Figure 7 and Supplementary Figures 3-5. Odor component indices are consistent with Table 3.
