# Supplementary material for: Antagonistic odor interactions in olfactory sensory neurons are widespread in freely breathing mice
Source: Nat Commun. 2020 Jul 3;11:3350. doi: 10.1038/s41467-020-17124-5 (PMC7335155; doi:10.1038/s41467-020-17124-5)
Supplement: Supplementary file 3 — Reporting Summary [file 41467_2020_17124_MOESM3_ESM.pdf]

nature research

Corresponding author(s): Venkatesh N. Murthy, Joseph D. Zak

Last updated by author(s): 06/3/2020

Reporting Summary

Nature Research wishes to improve the reproducibility of the work that we publish. This form provides structure for consistency and transparency in reporting. For further information on Nature Research policies, see Authors & Referees and the Editorial Policy Checklist .

Statistics

For all statistical analyses, confirm that the following items are present in the figure legend, table legend, main text, or Methods section.

n/a	Confirmed	
☐	☒	The exact sample size (n) for each experimental group/condition, given as a discrete number and unit of measurement
☐	☒	A statement on whether measurements were taken from distinct samples or whether the same sample was measured repeatedly
☐	☒	The statistical test(s) used AND whether they are one- or two-sided<br>*Only common tests should be described solely by name; describe more complex techniques in the Methods section.*
☐	☒	A description of all covariates tested
☐	☒	A description of any assumptions or corrections, such as tests of normality and adjustment for multiple comparisons
☐	☒	A full description of the statistical parameters including central tendency (e.g. means) or other basic estimates (e.g. regression coefficient) AND variation (e.g. standard deviation) or associated estimates of uncertainty (e.g. confidence intervals)
☐	☒	For null hypothesis testing, the test statistic (e.g. F, t, r) with confidence intervals, effect sizes, degrees of freedom and P value noted<br>*Give P values as exact values whenever suitable.*
☒	☐	For Bayesian analysis, information on the choice of priors and Markov chain Monte Carlo settings
☒	☐	For hierarchical and complex designs, identification of the appropriate level for tests and full reporting of outcomes
☐	☒	Estimates of effect sizes (e.g. Cohen's d, Pearson's r), indicating how they were calculated

Our web collection on statistics for biologists contains articles on many of the points above.

Software and code

Policy information about availability of computer code

Data collection	All data were collected using custom LabView code to control a multiphoton scan system and file writing.
Data analysis	Imaging data were processed using freely available packages including NoRmCorre v0.1.1 (https://github.com/flatironinstitute/NoRMCorre) and Caiman v1.4.1 (https://github.com/flatironinstitute/CaImAn). All analysis was performed using custom code written in Matlab2018a.

For manuscripts utilizing custom algorithms or software that are central to the research but not yet described in published literature, software must be made available to editors/reviewers. We strongly encourage code deposition in a community repository (e.g. GitHub). See the Nature Research guidelines for submitting code & software for further information.

Data

Policy information about availability of data

All manuscripts must include a data availability statement. This statement should provide the following information, where applicable:

- Accession codes, unique identifiers, or web links for publicly available datasets
- A list of figures that have associated raw data
- A description of any restrictions on data availability

All data and analysis code are available upon reasonable request to the corresponding author Venkatesh Murthy (vnmurthy@fas.harvard.edu).

Field-specific reporting

Please select the one below that is the best fit for your research. If you are not sure, read the appropriate sections before making your selection.

Life sciences study design

All studies must disclose on these points even when the disclosure is negative.

Sample size	Our experiments were designed to use 5-6 mice in each experimental group. The final numbers of mice were affected by surgical and imaging success rate. The number of mice was chosen so that statistics on imaging fields could be performed. From each animal multiple imaging fields can be used. Both glomeruli and individual sensory neurons were collected from the most optically dense imaging area in each animal. In some animals multiple imaging fields were used.
Data exclusions	Mice with unsuccessful surgeries or poor imaging quality are excluded. For experiments where odor mixture responses were predicted, predictions that yielded negative values were excluded. These criteria were pre-established because the kinetics of negative going fluorescence signals are difficult to interpret and differentiate from artifacts. Furthermore, the dynamic rage of negative GCaMP signals is very small thereby limiting our ability to yield useful interpretations of the data.
Replication	All experiments were at least performed in three or more mice for the reproducibility. The effects of mixture suppression could be observed across every animal and imaging field.
Randomization	All animals were of the same genotype in this study.
Blinding	Blinding was not necessary because there were no group comparisons in this study.

Reporting for specific materials, systems and methods

We require information from authors about some types of materials, experimental systems and methods used in many studies. Here, indicate whether each material, system or method listed is relevant to your study. If you are not sure if a list item applies to your research, read the appropriate section before selecting a response.

Materials & experimental systems

n/a	Involved in the study	
☒	☐	Antibodies
☒	☐	Eukaryotic cell lines
☒	☐	Palaeontology
☐	☒	Animals and other organisms
☒	☐	Human research participants
☒	☐	Clinical data

Methods

n/a	Involved in the study	
☒	☐	ChIP-seq
☒	☐	Flow cytometry
☒	☐	MRI-based neuroimaging

Animals and other organisms

Policy information about studies involving animals; ARRIVE guidelines recommended for reporting animal research

Laboratory animals	Mice were acquired from an in house breeding stock of OMP-GCaMP3 animals (Isogai et al., 2011). All surgeries and experiments were carried out in adult mice (6 weeks or older) of both sexes.
Wild animals	This study did not involve wild animals.
Field-collected samples	This study did not involve samples collected from the field.
Ethics oversight	All procedures were in accordance with protocols approved by the Institutional Animal Care and Use Committee at Harvard University.

Note that full information on the approval of the study protocol must also be provided in the manuscript.
